# Psychometric evaluation of the German version of the Recovering Quality of Life (ReQoL) measures in patients with affective disorders

**DOI:** 10.1007/s10198-022-01489-z

**Published:** 2022-07-01

**Authors:** Thomas Grochtdreis, Hans-Helmut König, Anju Devianee Keetharuth, Jürgen Gallinat, Alexander Konnopka, Holger Schulz, Martin Lambert, Anne Karow, Judith Dams

**Affiliations:** 1 Department of Health Economics and Health Services Research, Hamburg Center for Health Economics, University Medical Center Hamburg-Eppendorf, Hamburg, Germany; 2 School of Health and Related Research, The University of Sheffield, Sheffield, UK; 3 Department of Psychiatry and Psychotherapy, University Medical Center Hamburg-Eppendorf, Hamburg, Germany; 4 Department of Medical Psychology, University Medical Center Hamburg-Eppendorf, Hamburg, Germany

**Keywords:** Reliability, Validity, Responsiveness, EQ-5D, SF6D, Health-related quality of life, Mood disorders, Germany, I10, I30

## Abstract

**Background:**

The generic self-reported Recovering Quality of Life (ReQoL) measures were developed for measuring recovery-focused health-related quality of life (HrQoL) in persons with mental health conditions. The aim of this study was to assess the psychometric properties of the German version of the ReQoL measures in patients with affective disorders in Germany.

**Methods:**

Data from a patient sub-sample in a randomized controlled trial have been used (*N* = 393). The internal consistency and the test–retest reliability of the ReQoL measures were assessed using Cronbach’s Alpha and the intra-class correlation coefficient (ICC). The concurrent validity and the known-group validity of the ReQoL measures were assessed using Pearson’s Correlation coefficient and Cohen’s d. The responsiveness was assessed using Glass' Δ and the standardized response mean (SRM).

**Results:**

The reliability among the items of the ReQoL-20 was overall excellent. The ICC of the ReQoL-20 was *r* = 0.70, indicating moderate test–retest reliability. The concurrent validity of the ReQoL-20 with the clinical measure PHQ-9 was strong with a correlation coefficient of *r* = − 0.76. The known-group validity of the ReQoL-20 using PHQ-9 cut-off points was large with an effect size of *d* = 1.63. The ReQoL measures were sensitive to treatment response and remission of symptoms measured by the PHQ-9 with large effect sizes/SRM.

**Discussion:**

The psychometric properties of the ReQoL measures for the assessment of patients with affective disorders were overall good. With the ReQoL, valid and reliable measures for the assessment of recovery-focused HrQoL for persons with affective disorders are available in German language.

**Supplementary Information:**

The online version contains supplementary material available at 10.1007/s10198-022-01489-z.

## Introduction

The concept of health-related quality of life (HrQoL) includes the aspects of self-perceived well-being in relation to disease or treatment [1]. Currently, in economic evaluations of mental health care for patients with affective disorders in Germany, HrQoL is regularly assessed using generic patient reported-outcome measures (PROMs; e.g., [2–4]), with the EQ-5D [5, 6] and the Short Form health survey (SF-12) being most popular [7]. Generic PROMs have the advantage that they can be administered for a broad range of diseases and treatments, as they cover many aspects of self-perceived well-being [1].

Generic HrQoL measures, however, have the disadvantage that they are not sufficiently suitable to represent the recovery process of mental health conditions, such as affective disorders [8]. In cost-effectiveness analyses in Germany, it was regularly recognized that generic HrQoL measures may not be responsive to changes in symptom severity or self-efficacy (e.g., [2, 9, 10]). In the treatment of persons with mental health conditions, it is also increasingly recognized that these persons want to live a full life even when symptoms are present, and that this can contribute to the mental recovery process [11, 12]. Thus, to measure the recovery process, an instrument is needed that measures the impact on quality of life of persons with mental health conditions rather than symptoms [11, 13, 14]. For that reason, the Recovering Quality of Life (ReQoL) measures – ReQoL-10 and ReQoL-20 – have been developed to assess HrQoL for persons with mental health conditions and to capture the recovery process with respect to leading a meaningful life, even if symptoms of the mental health condition are present [11].

The ReQoL measures were developed on a broad theoretical basis and with the involvement of academics/psychometricians, policymakers, clinicians and patients [15]. The final 10/20 items of the measures were selected after factor analysis of data from more than 6500 patients and item response theory models employed to inform item selection. Recently, the ReQoL measures originally developed in English have been translated and linguistically validated in German using established methodology [16]. ReQoL measures are also available in other languages, such as Dutch [17], seven common Indian languages [18–24] and traditional Chinese [25].

The final ReQoL measures represent those components of recovery by the seven domains: activity, belonging and relationships, choice, control and autonomy, hope, self-perception, well-being and physical health [15, 26]. Furthermore, the items of the ReQoL measures are formulated negatively and positively and thereby are of decisive importance for measuring both improved and worsened HrQoL as a result of mental health problems [15, 26, 27]. Positively and negatively worded items are scored from zero to four and four to zero, respectively [15]. By summing up the scores of the items, an overall score for the ReQoL-10/ReQoL-20 can be calculated with zero representing the poorest HrQoL and 40/80 representing the highest HrQoL.

Furthermore, it is possible to calculate quality-adjusted life-years from the ReQoL measures for use in cost-utility analyses. For this purpose, preference weights from the general population in the United Kingdom were estimated and the ReQoL-Utility Index (UI) has been developed [28]. Unfortunately, preference weights from the general population in Germany are not available at this time.

So far, the psychometric properties of the ReQoL measures have been assessed in patients with anxiety and depression in the United Kingdom [29], in patients with psychosis in the Netherlands [17] and Singapore [30], in the general population in the United Kingdom [15] and Hong Kong [25] as well as in a convenience sample in the Netherlands [17]. For the patient populations, the ReQoL measures showed good internal consistency and better responsiveness and construct validity compared with the EQ-5D-5L in patients with depression, but not in patients with anxiety [29, 30]. Furthermore, the ReQoL-10 was reliable in a sample with patients with psychosis and the ReQoL measures showed good convergent and known-group validity [17]. In a first-episode psychosis population in Singapore, ReQoL-10 was found to have good internal consistency and adequate construct validity [30].

To the best of our knowledge, the psychometric properties of the ReQoL measures have not yet been assessed for patient populations in Germany. Therefore, the primary aim of this study was to assess the psychometric properties of the ReQoL-10 and ReQoL-20 for the assessment of patients with bipolar affective disorder, major depression and dysthymia in Germany. The secondary aim of this study was to assess the validity of the ReQoL-10 and ReQoL-20 by comparison with clinical measures and measures of HrQoL.

## Materials and methods

### Sample

Data used for this study were collected within a randomized controlled trial evaluating an evidence-based, stepped and coordinated care service model for mental disorders (RECOVER) [31]. Patients were recruited in the regular psychiatric care of the University Medical Center Hamburg-Eppendorf, Germany, from beginning of 2018 until the end of 2019. Patients were eligible for participation if they were at least at the age of 16 years and if they were diagnosed with at least one relevant mental disorder (among others, e.g. schizophrenic spectrum disorders, bipolar affective disorder, major depression, anxiety disorder or post-traumatic stress disorder) according to the International Statistical Classification of Diseases and Related Health Problems—10th Revision, German Modification (ICD-10) [31, 32].

Patients were excluded from the study if they fulfilled the criteria for organic mental disorders, addiction disorders as main diagnosis, and/or moderate to severe mental retardation. Furthermore, patients were excluded if they lacked correctable hearing and/or vision impairment and/or if they were with insufficient knowledge of German [31].

For the current study, the patient sample of the RECOVER trial was reduced to persons with bipolar affective disorder (ICD-10: F31), persons with major depression (ICD-10: F32.2) and persons with dysthymia (ICD-10: F34.1). Data was collected at baseline (*T*0), 6 months (*T*1) and 12 months after baseline (*T*2). The sample was further restricted to persons without missing information in the ReQoL measure at *T*0.

The trial was registered prospectively (NCT03459664), ethics approval was obtained from the ethics committee of the Hamburg Medical Association (PV5672) and all participants of the trial provided written informed consent. A detailed description of the RECOVER trial can be found elsewhere [31].

### Measures

The German 20-item version of the ReQoL measures (ReQoL-20) has been used for measuring recovery-focused HrQoL [16]. The first 10 items of the ReQoL-20 constitute the ReQoL-10. The 20 items are scored on a scale with five levels ranging from ‘none of the time’ to ‘most of the time’. In due consideration of the positive and negative wording of the items, the items scores were summed up to a total score ranging from 0 to 80, with higher scores indicating a higher recovery-focused HrQoL [15]. The ReQoL-10 was calculated from the first 10 items of the ReQoL-20 with a total score ranging from 0 to 40. While the ReQoL contains a physical health item, this is not included in the score [28].

Symptom severity was assessed by the German versions of the 9-question depression scale of the Patient Health Questionnaire (PHQ-9) for the measurement of depressive symptoms [33, 34] and the Altman Self-Rating Mania Scale (ASRM) for the assessment of the presence and severity of manic or hypomanic symptoms [35]. The social, occupational, and psychological functioning was assessed by the Global Assessment of Functioning (GAF) scale [36], and the severity of illness was assessed using the Clinical Global Impression – Severity scale (CGI-S) [37]. HrQoL was measured using the indexes of the German versions of the EQ-5D-5L [5, 6] based on German preference weights [38] and the SF-12 (SF-6D) [7, 39] based on preference weights from the United Kingdom [40] as well as the visual analogue scale of the EQ-5D-5L (EQ-VAS) [6]. Furthermore, the mental component summary score (MCS) and physical component summary score (PCS) were calculated from the respective mental and physical dimensions of the SF-12 [39]. Additional information on the constructs and scores of the measures used for the assessment of symptom severity and HrQoL are given in the Online Resource.

The sociodemographic variables self-reported age, sex, marital status, migration background, school-leaving qualification, and education were used for description of the sample. Furthermore, the number of comorbid DSM-IV diagnoses was collected and categorized into comorbid clinical disorders (axis I diagnoses) and personality disorders (axis II diagnoses) [41].

### Statistical analysis

Observations with missing information were removed from the analyses by casewise deletion. Sociodemographic characteristics of the sample were analyzed using descriptive statistics, and the distribution of the ReQoL-10/ReQoL-20 was assessed by histograms of the individual items for the total study sample. Normality of the distribution of the individual items of the ReQoL measures was analyzed using the Shapiro–Wilk test for normal data [42]. Reliability, validity, and responsiveness of the ReQoL measures were assessed. *T*0, *T*1 and *T*2 data was used to assess the test–retest reliability and the sensitivity to change of the ReQoL-10/ReQoL-20. All other analyses were based on *T*0 data.

The structural validity of the ReQoL measures was assessed using confirmatory factor analysis (CFA) to confirm a correlated traits model structure comprising of two distinct elements of positively and negatively worded items [26]. Goodness of fit was indicated by the root mean square error of approximation (RMSEA), the comparative fit index (CFI), and the Tucker-Lewis index (TLI) with cut-off values for the RMSEA and the CFI/TLI of  ≤ 0.08 and > 0.95, respectively [26, 43, 44]. Internal consistency, the extent to which scores of one measure are the same for repeated measurement using different sets of items, of the ReQoL measures was assessed between all halves of the questionnaires. Furthermore, test–retest reliability, the extent to which scores of one measure are the same for repeated measurement over time, of the ReQoL measures was assessed for the same measures applied over measurement time points. For the assessment of the internal consistency, the questionnaires were split in all possible halves and the average correlations of all halves were calculated using Cronbach’s Alpha [45]. Cronbach’s alpha coefficients above *α* ≥ 0.7 were defined as acceptable, above *α* ≥ 0.8 as good and above *α* ≥ 0.9 as excellent [46]. For the assessment of the test–retest reliability, only those persons without any improvement or worsening of symptoms between measurement points were selected from the sample. Unchanged symptoms between measurement points were defined as PHQ-9 (ASRM) difference < $\left|5\right|$ ($\left|4\right|$) between measurement points [35, 47]. Test–retest reliability was calculated using the intra-class correlation coefficient (ICC) [48]. ICC below *r* = 0.50 were defined as poor, between *r* = 0.50 and *r* = 0.75 as moderate, between *r* = 0.75 and *r* = 0.90 as good, and above *r* = 0.90 as excellent [49].

The concurrent validity, the extent to how well one measure compares to other measures, of the ReQoL measures was assessed by comparison with clinical measures (PHQ-9 and ASRM) and measures of HrQoL (EQ-5D-5L, EQ-VAS, SF-6D, MCS and PCS). Furthermore, the known-group validity, the extent to how well one measure can demonstrate different scores for different groups, was assessed by comparison of scores for groups with less or more severe symptoms, and with good or poor global functioning and lower or higher severity of illness. The concurrent validity was assessed using Pearson’s Correlation Coefficient (PCC), and by scatterplots and LOWESS curves of the respective scores. PCC below *r* = $\left|0.30\right|$ were defined as negligible, between *r* = $\left|0.30\right|$ and *r* = $\left|0.50\right|$ as low, between *r* = $\left|0.50\right|$ and *r* = $\left|0.70\right|$ as moderate, between *r* = $\left|0.70\right|$ and *r* = $\left|0.90\right|$ as high and above* r* = $\left|0.90\right|$ as very high [50]. For the assessment of the known-group validity, the sample was split into groups with good and poor health using generic measures (CGI-S > 4, GAF ≤ 50 [36, 37]) and with less or more severe symptoms using clinical measures (PHQ-9 > 4 and ASRM ≤ 50 [33–35]). The known-group validity was assessed, in accordance with the psychometric evaluation of the original ReQoL measures [15], using the effect size (ES) Cohen’s *d*. ES of *d* = 0.20 were defined as small, of *d* = 0.50 as medium and of *d* = 0.80 as large [51, 52].

Responsiveness, the ability of one measure to detect a significant change assessed using a gold standard, of the ReQoL measures was assessed by sensitivity to treatment response (reduction of the PHQ-9 by ≥ 5 points) and remission of symptoms (PHQ-9 < 5) between time points. Sensitivity to change was assessed using the ES Glass' Δ and the standardized response mean (SRM). Furthermore, receiver operating characteristics (ROC) curves were constructed and discriminative abilities were assessed using the area under the curve (AUC), with an AUC of 1.0 defined as perfect discriminative abilities and an AUC of 0.5 defined as random chance. Optimal cut-off values were determined by the distance to the top-left corner from points on the ROC curve. Distance was defined as *d*^2^ = (1 − sensitivity)^2^ + (1 − specificity)^2^. Thereby, the point with the lowest distance was defined as cut-off point [53].

Data analyses were performed using Stata/MP 17.0 (StataCorp, TX, USA). All statistics were two-sided with a significance level of *p* < 0.05.

## Results

### Sample characteristics

The mean age of the total sample of persons with mood disorders (*n* = 393) was 39 years (Table 1). More than half of the sample was female (56%). The majority of the sample was single (51%), had an upper secondary school certificate (56%) and had a vocational training degree (45%). The mean number of comorbid DSM-IV axis I diagnoses and axis II diagnoses of the sample was 0.74 and 0.11, respectively. The complete sociodemographic characteristics of the total sample and the sub-samples are shown in Table 1.

**Table 1:** Sociodemographic characteristics of persons with mood disorders (F30-F39; *n* = 393)

Sociodemographic characteristic	All Persons with mood disorders (F30-F39; *n* = 393)^b^	Persons with bipolar affective disorder (F31; *n* = 36)	Persons with major depression (F32.2; *n* = 350)	Persons with dysthymia (F34.1; *n* = 6)
Age: Mean (SD)	39.18 (14.93)	40.03 (13.95)	39.07 (15.00)	38.83 (19.92)
Female sex: *n* (%)	220 (55.98)	22 (61.11)	194 (55.43)	3 (50.00)
With migration background^a^: *n* (%)	119 (30.43)	15 (41.67)	101 (29.02)	2 (33.33)
Marital status: *n* (%)				
Single	200 (50.89)	23 (63.89)	172 (49.14)	5 (83.33)
Married	60 (15.27)	6 (16.67)	54 (15.43)	–
In partnership	100 (25.45)	6 (16.67)	93 (36.57)	–
Divorced/widowed	33 (8.40)	1 (2.78)	31 (8.86)	1 (16.67)
School-leaving qualification: *n* (%)				
No school-leaving qualification	9 (2.29)	–	9 (2.57)	–
Lower secondary school certificate	160 (40.71)	5 (13.89)	150 (42.86)	4 (66.67)
Upper secondary school certificate	219 (55.73)	31 (86.11)	186 (53.14)	2 (33.33)
Other school-leaving qualification	5 (1.27)	–	5 (1.43)	–
Education: *n* (%)				
No completed education	111 (28.46)	11 (30.56)	97 (27.95)	2 (33.33)
Vocational training degree	175 (44.87)	9 (25.00)	163 (46.97)	3 (50.00)
University degree/master craftsman's examination	104 (26.67)	19 (44.44)	87 (25.07)	1 (16.67)
Number of comorbid DSM-IV diagnoses: mean (SD)				
Axis I diagnoses	0.74 (0.95)	0.67 (0.96)	0.74 (0.95)	0.17 (0.52)
Axis II diagnoses	0.11 (0.37)	0.14 (0.36)	0.11 (0.37)	0.33 (0.41)

*SD* standard deviation

^a^Person had an own migration experience or was born to at least one parent with own migration experience

^b^One person had an unspecified mood disorder (F39)

### Distribution of scores

The mean scores of the ReQoL-20 and ReQoL-10 in the total sample were 33.07 (SD 13.31) and 16.61 (SD 6.98) at *T*0, with a range from 8 to 76 and 3 to 39, respectively. The scores of both the ReQoL-20 and ReQoL-10 were slightly right skewed and leptokurtic with no apparent floor and ceiling effects (data not shown). At *T*1, the mean scores were 44.17 (SD 16.75) and 22.35 (SD 8.82) and at *T*2, the mean scores were 45.52 (SD 16.36) and 23.01 (SD 8.71). In the sub-samples of persons with bipolar affective disorder and of persons with major depression and dysthymia, the mean scores of the ReQoL-20 were 42.08 (SD 16.64) and 32.19 (SD 12.61) at *T*0, 48.00 (SD 15.77) and 43.71 (SD 16.86) at *T*1, and 48.93 (SD16.12) and 45.13 (SD 16.37) at *T*2, respectively. The respective mean scores of the ReQoL-10 were 21.44 (8.63) and 16.13 (6.61) at *T*0, 24.40 (8.17) and 22.11 (8.88) at *T*1, and 25.20 (SD 8.23) and 22.77 (SD 8.74) at *T*2.

**Fig. 1:**
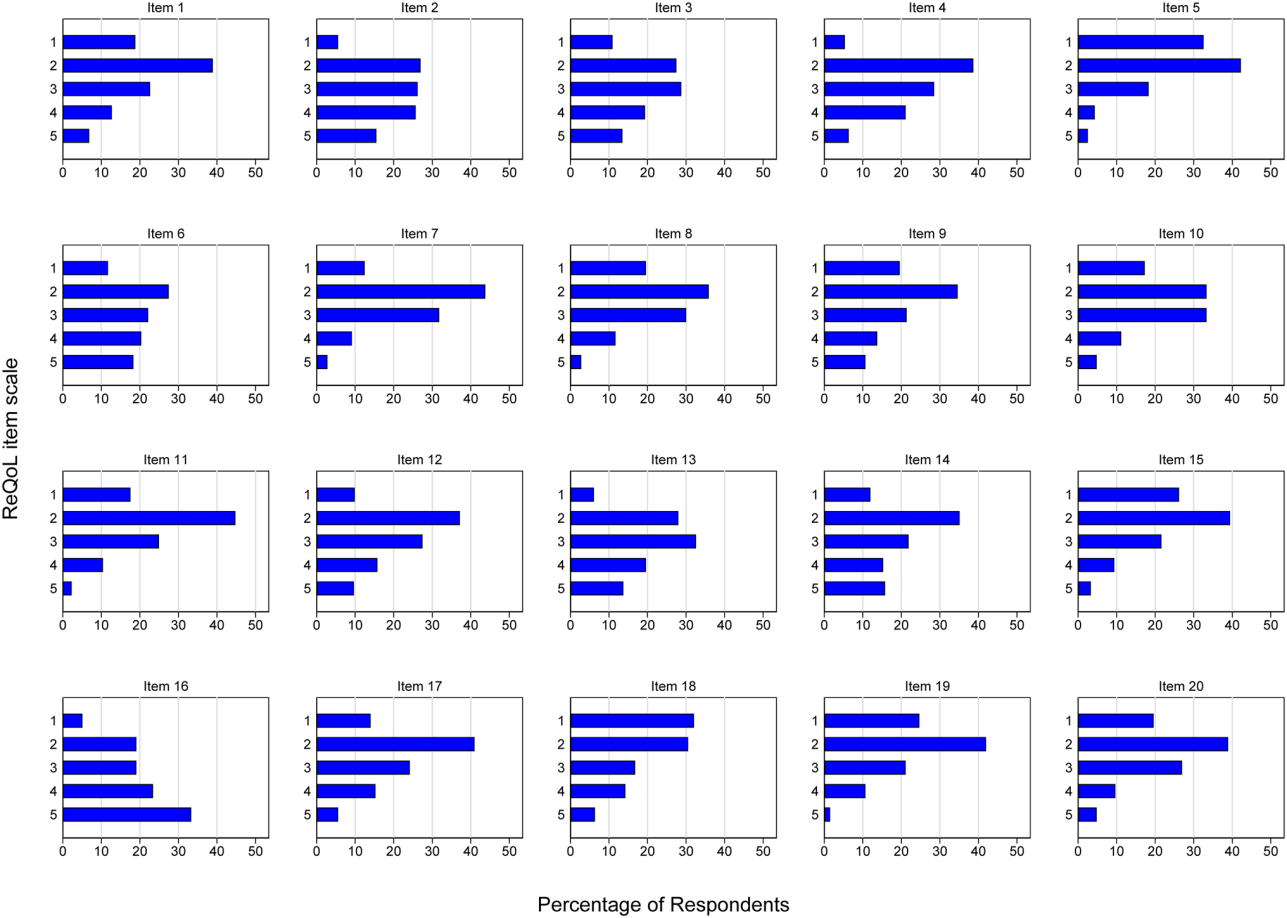
Distribution of the individual items of the ReQoL measures at *T*0 (*n* = 393). *1* None of the time, *2* only occasionally, *3* sometimes, *4* often, *5* most or all of the time, *item 1* I found it difficult to get started with everyday tasks, *item 2* I felt able to trust others, *item 3* I felt unable to cope, *item 4* I could do the things I wanted to do, *item 5* I felt happy, *item 6* I thought my life was not worth living, *item 7* I enjoyed what I did, *item 8* I felt hopeful about my future, *item 9* I felt lonely, *item 10* I felt confident in myself, *item 11* I did things I found rewarding, *item 12* I avoided things I needed to do, *item 13* I felt irritated, *item 14* I felt like a failure, *item 15* I felt in control of my life, *item 16* I felt terrified, *item 17* I felt anxious, *item 18* I had problems with my sleep, *item 19* I felt calm, *item 20* I found it hard to concentrate

The majority of the individual items of the ReQoL measures at T0 were not normally distributed (all with *p* < 0.05), with the exception of the items 2 (‘I felt able to trust others’), 3 (‘I felt unable to cope’), 6 (‘I thought my life was not worth living’), 10 (‘I felt confident in myself’) and 13 (‘I felt irritated’; Fig. 1). The three items of the ReQoL measures with the most positively skewed distribution were the items 5 (‘I felt happy’), 15 (‘I felt in control of my life’) and 18 (‘I had problems with my sleep’). Goodness of fit of the correlated traits model structure of the ReQoL-20 (ReQoL-10) was confirmed with a RMSEA of 0.08 (0.09), yet with a CFI of 0.87 (0.92) and a TLI of 0.85 (0.89).

### Reliability

Cronbach’s’ alpha for the ReQoL-20 (ReQoL-10) in the total sample with mood disorders was *α* = 0.91 (*α* = 0.83), indicating excellent (good) reliability among the items. Cronbach’s alpha for the ReQoL-20 (ReQoL-10) in the sub-samples of persons with bipolar affective disorder, and of persons with major depression and dysthymia was *α* = 0.94 (*α* = 0.87) and *α* = 0.89 (*α* = 0.81), respectively, indicating excellent (good) and good reliability among the items.

The ICC of the ReQoL-20 (ReQoL-10) in the total sample with mood disorders without improvement or worsening of symptoms from *T*0 to *T*1 and *T*1 to *T*2 measured by the PHQ-9 was *r* = 0.70 (*r* = 0.68) and *r* = 0.76 (*r* = 0.75), indicating moderate and good test–retest reliability, respectively (Table 2).

**Table 2:** Test–Retest Reliability of the ReQoL-10 and ReQoL-20 of persons without improvement or worsening of symptoms between measurement points for persons with mood disorders

			All persons with mood disorders (F30-F39)^c^		Persons with bipolar affective disorder (F31)		Persons with major depression and dysthymia (F32.2 and F34.1)	
			*N* (%)	ICC	*N* (%)	ICC	*N* (%)	ICC
*T*0 to *T*1	ReQoL-10	PHQ-9^a^ (*n* = 282)	139 (49.29)	0.68	13 (48.15)	0.59	126 (49.61)	0.68
		ASRM^b^ (*n* = 31)	–		19 (61.29)	0.45	–	–
	ReQoL-20	PHQ-9^a^ (*n* = 282)	139 (49.29)	0.75	13 (48.15)	0.72	126 (49.61)	0.75
		ASRM^b^ (*n* = 31)	–	–	19 (61.28)	0.43	–	–
*T*1 to *T*2	ReQoL-10	PHQ-9^a^ (*n* = 252)	191 (75.79)	0.84	22 (78.57)	0.78	169 (75.45)	0.85
		ASRM^b^ (*n* = 28)	–	–	18 (64.29)	0.81	–	–
	ReQoL-20	PHQ-9^a^ (*n* = 252)	191 (75.79)	0.86	22 (78.57)	0.78	169 (75.45)	0.87
		ASRM^b^ (*n* = 28)	–	–	18 (64.29)	0.78	–	–
*T*0 to *T*2	ReQoL-10	PHQ-9^a^ (*n* = 284)	130 (45.78)	0.70	13 (44.83)	0.74	117 (45.88)	0.68
		ASRM^b^ (*n* = 29)	–	–	16 (55.17)	0.47	–	–
	ReQoL-20	PHQ-9^a^ (*n* = 284)	130 (45.78)	0.76	13 (44.83)	0.82	117 (45.88)	0.75
		ASRM^b^ (*n* = 29)	–	–	16 (55.17)	0.39	–	–

*ICC* Intra-class correlation coefficient

^a^PHQ-9: missing improvement or worsening of symptoms between measurement points was defined as − 5 < PHQ-9 difference < 5

^b^ASRM: missing improvement or worsening of symptoms between measurement points was defined as − 4 < ASRM difference < 4

^c^One person had an unspecified mood disorder (F39)

### Validity

The correlation coefficient between the ReQoL-20 and the ReQoL-10 was *r* = 0.94. In the sub-samples of persons with bipolar affective disorder and of persons with major depression and dysthymia, the correlation coefficient was *r* = 0.96 and *r* = 0.93, respectively.

The concurrent validity of the ReQoL-20 and ReQoL-10 with the clinical measure PHQ-9 was strong and moderate, indicated by a correlation coefficient of *r* =  − 0.76 and *r* =  − 0.69, respectively (Table 3, Figures S1 and S2 in the Online Resource). In the sub-samples of persons with bipolar affective disorder and of persons with major depression and dysthymia, the correlation coefficients between the ReQoL-20 (ReQoL-10) and the PHQ-9 were *r* =  − 0.80 (*r* =  − 0.70) and *r* =  − 0.75 (*r* =  − 0.67), indicating an overall strong negative linear relationship.

**Table 3:** Concurrent validity between ReQoL-10/ReQoL-20, clinical measures (PHQ-9, ASRM) and measures of health-related quality of life (EQ-5D-5L, EQ-VAS, SF-6D, MCS, PCS) for persons with mood disorders

PCC		All persons with mood disorders (F30-F39; *n* = 393^a^)	Persons with bipolar affective disorder (F31; *n* = 36)	Persons with major depression and dysthymia (F32.2 and F34.1; *n* = 356)
ReQoL-10	PHQ-9 (*n* = 383)	− 0.69	− 0.70	− 0.67
	ASRM (*n* = 36)	–	0.53	–
	EQ-5D-5L (*n* = 384)	0.55	0.55	0.54
	EQ-5D-5L domain anxiety/depression (*n* = 387)	− 0.64	− 0.64	− 0.62
	EQ-VAS (*n* = 391)	0.56	0.76	0.53
	SF-6D (*n* = 358)	0.62	0.74	0.58
	MCS (*n* = 349)	0.63	0.78	0.57
	PCS (*n* = 349)	0.29	0.44	0.29
ReQoL-20	PHQ-9 (*n* = 383)	− 0.76	− 0.80	− 0.75
	ASRM (*n* = 36)	–	0.52	–
	EQ-5D-5L (*n* = 384)	0.57	0.53	0.56
	EQ-5D-5L domain anxiety/depression (*n* = 387)	− 0.66	− 0.61	− 0.65
	EQ-VAS (*n* = 391)	0.56	0.72	0.53
	SF-6D (*n* = 358)	0.63	0.70	0.60
	MCS (*n* = 349)	0.63	0.69	0.60
	PCS (*n* = 349)	0.29	0.46	0.29

All correlation coefficients were statistically significant with *p* ≤ 0.001

*PCC* Pearson’s Correlation Coefficient

^a^One person had an unspecified mood disorder (F39)

The concurrent validity of the ReQoL-20 and ReQoL-10 with measures of HrQoL was overall moderate, with correlation coefficients ranging from *r* = 0.55 to *r* = 0.63 (Table 3, Figures S3 and S4 in the Online Resource). The only exception was the concurrent validity of the ReQoL-20 and ReQoL-10 with the PCS, which was only weak with a correlation coefficient of *r* = 0.29. All observed correlations were in the expected directions. The correlation coefficients between further measures of HrQoL and clinical measures are given in Table S4 in the Online Resource.

The mean scores of the ReQoL-20 (ReQoL-10) in the sample with minimal to moderate depression and moderately severe depression to severe depression measured by the PHQ-9 were 42.67 (21.16) and 25.70 (13.13), respectively (Table S2 in the Online Resource). The corresponding known-group validity of the ReQoL-20 and ReQoL-10 using PHQ-9 cut-off points was large with ES of *d* = 1.63 and *d* = 1.39, respectively (Table 4).

**Table 4:** Known-group validity of ReQoL-10/ReQoL-20 using cut-off points of clinical measures (PHQ-9^a^, ASRM^b^) as well as generic measures (CGI-S^c^, GAF^d^)

Cohen’s D		All persons with mood disorders (F30-F39; *n* = 393^e^)	Persons with bipolar affective disorder (F31; *n* = 36)	Persons with major depression and dysthymia (F32.2 and F34.1; *n* = 356)
ReQoL-10	PHQ-9 (*n* = 383)	1.39	1.65	1.34
	ASRM (*n* = 36)	–	− 1.09	–
	CGI-S (*n* = 393)	0.37	0.14	0.63
	GAF (*n* = 393)	0.38	0.31	0.56
ReQoL-20	PHQ-9 (*n* = 383)	1.63	1.97	1.58
	ASRM (*n* = 36)	–	− 1.11	–
	CGI-S (*n* = 393)	0.36	0.09	0.59
	GAF (*n* = 393)	0.36	0.19	0.56

^a^PHQ-9 was dichotomized into minimal to moderate depression (PHQ ≤ 14) vs. moderately severe depression to severe depression (PHQ-9 > 14)

^b^ASRM was dichotomized into low probability of a manic or hypomanic condition (ASRM < 6) vs. high probability of a manic or hypomanic condition (ASRM ≥ 6)

^c^The seven original categories of the CGI-S were dichotomized into less severe (1 ≤ CGI-S ≤ 4) vs. more severe illness (4 > CGI-S ≤ 7)

^d^The GAF was dichotomized into good (GAF > 50) vs. poor global functioning (GAF ≤ 50)

^e^One person had an unspecified mood disorder (F39)

The mean scores of the ReQoL-20 (ReQoL-10) in the sample with good or poor global functioning dichotomized by the GAF were 35.10 (17.68) and 30.35 (15.16), respectively (Table S2 in the Online Resource). When the severity of illness was dichotomized into lower or higher by the CGI-S, the mean scores of the ReQoL-20 (ReQoL-10) were 34.78 (17.57) and 30.07 (14.92). The corresponding known-group validity of the ReQoL-20 and ReQoL-10 using CGI-S and GAF cut-off points was small with ES ranging from *d* = 0.36 to *d* = 0.38.

### Responsiveness

The ReQoL measures were sensitive to treatment response measured by the PHQ-9. The ES/SRM of the ReQoL-20 and ReQoL-10 ranged between 1.20/1.40 and 2.02/1.73 between all measurement points, indicating high responsiveness (Table 5, Table S3 in the Online Resource). The mean difference of the ReQoL-20 (ReQoL-10) in the sample with treatment response was 22.11 (10.70) between *T*0 and *T*1, and 16.58 (8.35) between *T*1 and *T*2. Mean differences and ES/SRM of measures of HrQoL based on treatment response measured by the PHQ-9 are given in Table S4 in the Online Resource.

**Table 5:** Sensitivity to change of ReQoL-10/ReQoL-20 based on the clinical measure PHQ-9 for persons with mood disorders—Treatment response^a^

		All persons with mood disorders (F30-F39)			Persons with bipolar affective disorder (F31)			Persons with major depression and dysthymia (F32.2 and F34.1)		
		*N* (%)	Mean difference (SD)	ES/SRM	*N* (%)	Mean difference (SD)	ES/SRM	*N* (%)	Mean difference (SD)	ES/SRM
*T*0 to *T*1	ReQoL-10 (*n* = 282)	132 (46.81)	10.70 (7.60)	1.73/1.40	9 (33.33)	11.00 (6.12)	1.43/1.80	122 (48.03)	10.64 (7.73)	1.75/1.38
	ReQoL-20 (*n* = 282)		22.11 (14.58)	1.90/1.52		26.00 (14.83)	1.82/2.35		21.75 (14.83)	1.89 /1.47
*T*1 to *T*2	ReQoL-10 (*n* = 263)	40 (15.21)	8.35 (5.87)	1.20/1.48	3 (10.34)	12.00 (8.72)	1.04/1.33	37 (15.81)	8.05 (5.65)	1.21/1.53
	ReQoL-20 (*n* = 263)		16.58 (11.16)	1.25/1.42		25.67 (19.35)	1.06/1.38		15.84 (10.33)	1.26/1.43
*T*0 to *T*2	ReQoL-10 (*n* = 284)	133 (46.83)	11.31 (7.26)	1.76/1.73	9 (31.03)	13.44 (5.94)	1.93/1.94	124 (48.63)	11.15 (7.34)	1.74/1.71
	ReQoL-20 (*n* = 284)		22.98 (13.29)	2.02/1.56		28.11 (14.50)	2.73/2.26		22.60 (13.19)	1.97/1.52

The effect size was calculated by dividing the mean change on the ReQoL-10/ReQoL-20 by the standard deviation of the ReQoL-10/ReQoL-20 at baseline (Glass' Δ). The standardized response mean was calculated by dividing the mean change on the ReQoL-10/ReQoL-20 by the standard deviation of the change

*ES* Effect size, *SRM* Standardized response mean

^a^Treatment response was defined as reduction of the PHQ-9 by ≥ 5 points

**Fig. 2:**
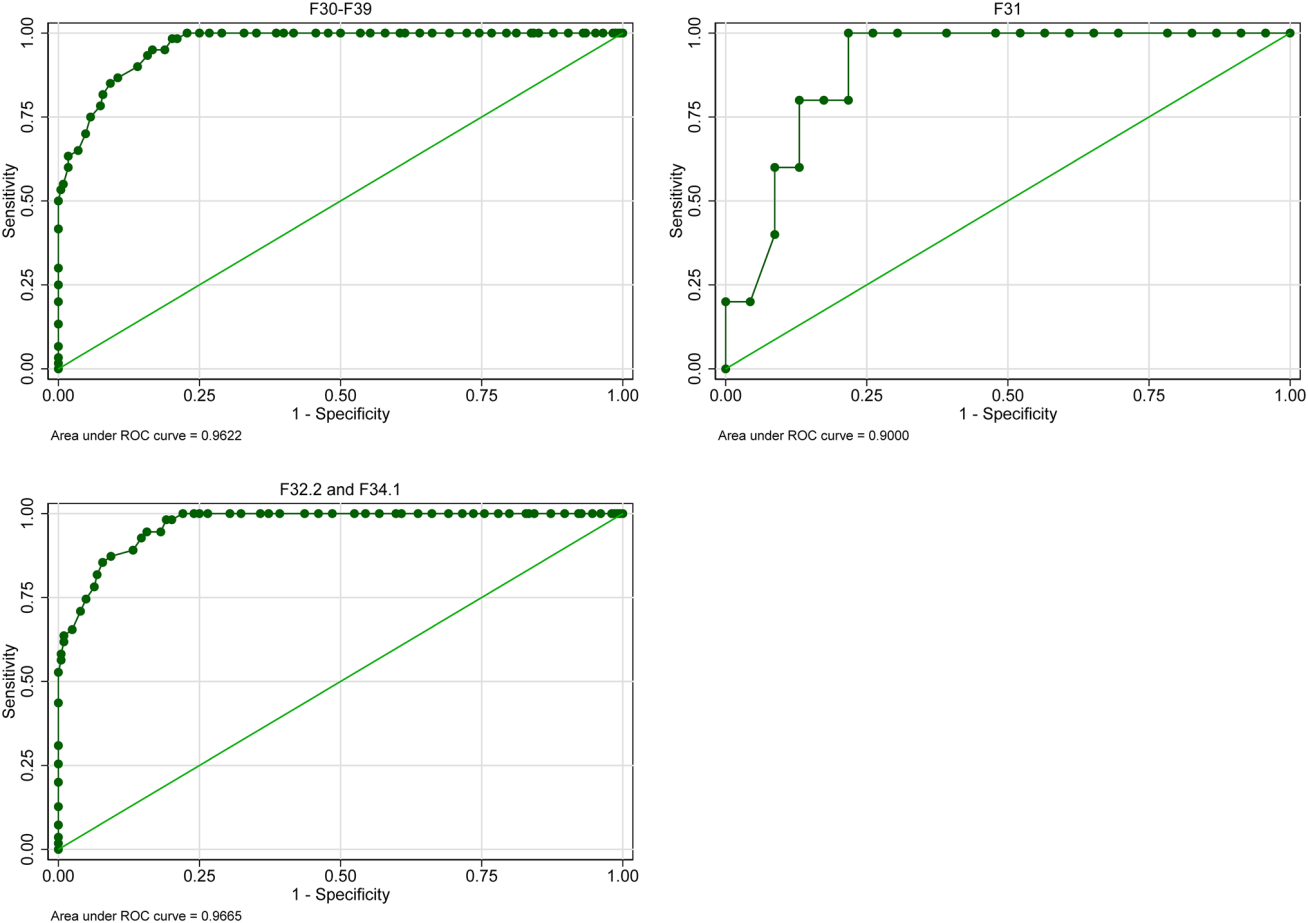
ROC curves of the ReQoL-20 difference between *T*0 and *T*1 and treatment response based on the clinical measure PHQ-9 for persons with mood disorders (*n* = 282)

ROC analyses showed AUC for ReQoL-20 and ReQoL-10 differences and treatment response measured by the PHQ-9 ranging from 0.87 to 0.89 and from 0.84 to 0.85, respectively (Fig. 2, Figure S3 and Table S5 in the Online Resource). The optimal cut-off value for treatment response was a ReQoL-20 (ReQoL-10) difference ≥ 12 (≥ 6) with a sensitivity of 78.03% (75.76%) and a specificity of 85.26% (80.77%).

For remission of symptoms measured by the PHQ-9, the ReQoL measures were also sensitive with large ES/SRM of the ReQoL-20 and ReQoL-10 between *T*0 and *T*1 (1.64/1.49 and 1.64/1.51) and with moderate ES/SRM between *T*1 and *T*2 (0.45/0.56 and 0.47/0.53; Table 6, Table S3 in the Online Resource). The mean difference of the ReQoL-20 (ReQoL-10) in the sample with remission of symptoms was 24.65 (12.13) between *T*0 and *T*1, and 5.80 (3.12) between *T*1 and *T*2. Mean differences and ES/SRM of measures of HrQoL based on remission of symptoms measured by the PHQ-9 are given in Table S6 in the Online Resource.

**Table 6:** Sensitivity to change of ReQoL-10/ReQoL-20 based on the clinical measure PHQ-9 for persons with mood disorders—Remission of symptoms^a^

		All persons with mood disorders (F30-F39)			Persons with bipolar affective disorder (F31)			Persons with major depression and dysthymia (F32.2 and F34.1)		
		*N* (%)	Mean diff. (SD)	ES/SRM	*N* (%)	Mean diff. (SD)	ES/SRM	*N* (%)	Mean diff. (SD)	ES/SRM
*T*0 to *T*1	ReQoL-10 (*n* = 282)	60 (21.28)	12.13 (8.04)	1.64/1.51	5 (18.52)	12.20 (8.11)	1.44/1.51	55 (21.65)	12.13 (8.11)	1.64/1.49
	ReQoL-20 (*n* = 282)		24.65 (16.55)	1.64/1.49		25.8 (13.12)	1.61/1.97		24.55 (16.93)	1.63/1.45
*T*1 to *T*2	ReQoL-10 (*n* = 263)	64 (24.33)	3.12 (5.54)	0.47/0.53	7 (24.14)	4.14 (9.04)	0.54/0.47	57 (24.36)	3.00 (5.08)	0.45/0.56
	ReQoL-20 (*n* = 263)		5.80 (10.88)	0.45/0.56		9.14 (19.29)	0.59/0.46		5.41 (9.62)	0.42/0.59
*T*0 to *T*2	ReQoL-10 (*n* = 284)	64 (22.54)	12.33 (8.72)	1.48/1.46	7 (24.14)	9.00 (11.08)	0.89/0.87	57 (22.35)	12.73 (8.43)	1.57/1.57
	ReQoL-20 (*n* = 284)		24.58 (16.82)	1.53/1.41		21.71 (25.07)	1.04/0.81		24.92 (15.85)	1.60/1.51

The effect size was calculated by dividing the mean change on the ReQoL-10/ReQoL-20 by the standard deviation of the ReQoL-10/ReQoL-20 at baseline (Glass' Δ). The standardized response mean was calculated by dividing the mean change on the ReQoL-10/ReQoL-20 by the standard deviation of the change

*ES* Effect size, *SRM* Standardized response mean.

^a^Remission of symptoms was defined as a PHQ-9 score of < 5

**Fig. 3:**
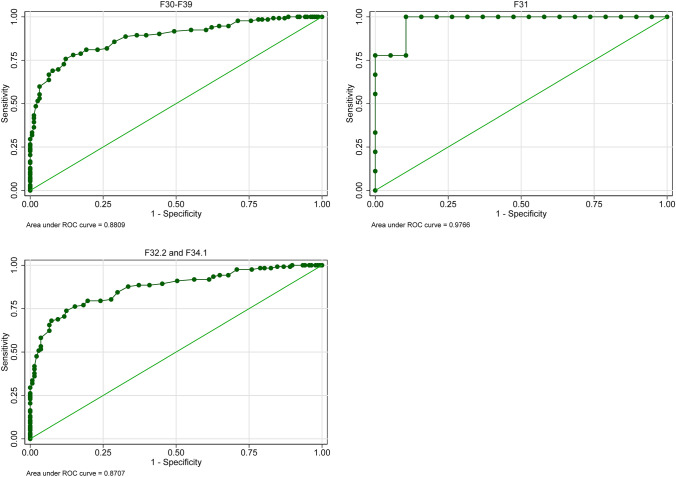
ROC curves of the ReQoL-20 at *T*1 and remission of symptoms based on the clinical measure PHQ-9 for persons with mood disorders (*n* = 282)

ROC analyses showed AUC for ReQoL-20 (ReQoL-10) differences and remission of symptoms measured by the PHQ-9 of 0.96 (0.94) at *T*1 and 0.83 (0.81) and *T*2 (Fig. 3, Figure S4 and Table S7 in the Online Resource). The optimal cut-off value for remission of symptoms was a ReQoL-20 (ReQoL-10) score of 57 (30) with a sensitivity of 86.67% (85.00%) and a specificity of 89.47% (89.04%).

## Discussion

This study aimed to assess the psychometric properties of the ReQoL measures in patients with affective disorders in Germany. The reliability of the ReQoL measures in this sample was overall good. The internal consistency was good to excellent and the test–retest reliability was moderate to good. The concurrent validity of the ReQoL measures with the clinical measure PHQ-9 and measures of HrQoL was strong and moderate, respectively. The ReQoL measures were able to distinguish between samples with minimal to moderate depression and moderately severe to severe depression measured by the PHQ-9 with large ES. The ReQoL measures, however, had a small known-group validity when the global functioning and the severity of illness was dichotomized into good/poor and lower/higher by the GAF and the CGI-S, respectively. Also, the ReQoL measures were sensitive to treatment response and remission of symptoms measured by the PHQ-9 with large ES.

The assessment of the psychometric properties of the ReQoL measures in a UK sample of patients with anxiety and depression showed similar results [29]. The concurrent validity of the ReQoL-10 with the clinical measure PHQ-9 was only moderate and the concurrent validity of the ReQoL-10 with the HrQoL measure EQ-5D-5L was low. The known-group validity of the ReQoL-10, however, was large, just as in the current sample. Furthermore, for remission of symptoms measured by the PHQ-9, the ReQoL-10 was responsive also with a large ES [29]. For patients with psychosis in the Netherlands, the psychometric properties of the ReQoL measures were also in line with the results of the current study [17]. The reliability of the ReQoL-10 was good and the concurrent validity of the ReQoL-10 with the HrQoL measure EQ-5D-5L was moderate. Furthermore, the known-group validity of the ReQoL measures was large for groups with lower and higher depression severity [17]. The structural validity of the ReQoL measures in the current sample using a correlated traits model was acceptable, yet comparably worse than the structural validity of the bi-factor model of the ReQoL measures in samples of the UK and Singapore, which had lower RMSEA (0.05 and 0.07 vs. 0.08) [26, 30].

The mean total ReQoL-10 score in the current study was lower than the mean scores of the patient samples reported in the other studies (16.6 vs. 18.6 to 27.8) [17, 29, 30]. One reason for this comparatively low mean total ReQoL-10 score could be the hospital setting from which recruitment for the sample of the current study took place [31]. By contrast, the settings of the other studies were outpatient care [29] or ongoing care [17]. Other reasons might be differences in recovery-focused HrQoL between patients with affective disorders and patients with anxiety [29, 30] or psychosis [17], or between patients of different countries.

Both ReQoL measures were found to be valid and reliable for the assessment of recovery-focused HrQoL for persons with affective disorders. However, the ReQoL-10 was less reliable with only good reliability among its items compared to the ReQoL-20 (*α* = 0.83 vs. *α* = 0.91). Furthermore, the concurrent validity of the ReQoL-10 with the PHQ-9 was only moderate, although the known-group validity and the responsiveness was comparably large for both ReQoL measures. In conjunction with the very high correlation between the ReQoL-10 and the ReQoL-20 (*r* = 0.94) it may be suggested that the ReQoL-10 is sufficient for the assessment of recovery-focused HrQoL for persons with affective disorders. Yet, the ReQoL-20 might be of use to provide a complete picture of the recovery process in research and psychiatric regular care [15]. Furthermore, compared with the further measures of HrQoL, the ReQoL-20 had a high concurrent validity with the PHQ-9 (PCC = − 0.76), whereas the concurrent validity of the EQ-5D-5L (PCC =  − 0.58) and the SF-6D (PCC =  − 0.55) was only moderate.

Large ES of the ReQoL measures for both, treatment response and remission of symptoms, show that the ReQoL measures are a sensitive and responsive measure for use in the area of affective disorders. With regard to the measures of HrQoL, it can be stated that the EQ-5D-5L was overall sensitive and responsive, yet with only moderate ES for both treatment response and remission of symptoms. Furthermore, the EQ-5D-5L index scores were extremely skewed compared with the distribution of the ReQoL-measure scores, with 3.39% of the sample indicating no problems in any dimension. Contrary to the EQ-5D-5L, the ES of the SF-6D were overall large and the distribution of its scores were only slightly skewed. Hence, the SF-6D can be considered a sensitive and responsive measure for use in the area of affective disorders.

As the EQ-5D was also previously shown to be possibly not suitable for use in the affective disorders and other mental health areas due to low responsiveness [8], the ReQoL measures and especially the ReQoL-UI derived from this may be promising for the concurrent use in mental health-related cost-utility analyses in Germany [28, 29]. The ReQoL measures may be of great importance for resource allocation decisions related to mental health services [15], as they are not only able to measure recovery-focused HrQoL but also change in depressive symptom severity and also possibly change in symptom severity of other mental health problems.

### Limitations

There are several limitations to this study. First, for the psychometric evaluation of the ReQoL measures, a patient sample of a randomized controlled trial has been used that has been recruited in a university clinical setting from catchment area of the University Medical Center Hamburg-Eppendorf, Hamburg. For this reason, the generalizability of the psychometric evaluation may be limited to inpatients with mood disorders. Second, for the analysis, only complete cases have been used, resulting in a loss of information and potentially introducing bias to the results of this study. Yet, sample size for the psychometric evaluation was large and a variety of clinical measures, generic measures and measures of HrQoL was available for comparison. Third, both ReQoL measures were assessed simultaneously within one person. Ideally, study participants would have been randomly assigned to one of the two measures. However, a larger sample would have been necessary for this purpose. Fourth, a correlated traits model structure comprising of two distinct elements of positively and negatively worded items was obtained for the CFA. However, a bi-factor model structure of the ReQoL measures was confirmed elsewhere [26, 30]. Unfortunately, it was not possible to fit a bi-factor CFA model to the current sample, as convergence was not achieved. Last, test–retest reliability was assessed over a period of 6 months. A shorter period of time between test and retest would have been more suitable, as this would ensure no change in symptom severity would have been occurred [54, 55]. Yet, for the assessment of the test–retest reliability, only those persons without any improvement or worsening of symptoms between measurement points were selected from the sample to minimize the problem of a long assessment period. Notwithstanding, the test–retest reliability of the ReQoL measures for patients with affective disorders should be interpreted with caution.

### Conclusion

The German version of the ReQoL measures is valid and reliable for the assessment of recovery-focused HrQoL for persons with affective disorders. As they have proven to also measure change in depressive symptom severity, the ReQoL measures are promising for use in mental HrQoL research as well as health economic research. Further research is needed in order to estimate preference weights from the general population for the development of a German ReQoL-UI.

## Supplementary Information

Below is the link to the electronic supplementary material.

Supplementary file1 (DOCX 467 KB)
