# Supplementary material for: Psychometric evaluation of the German version of the Recovering Quality of Life (ReQoL) measures in patients with affective disorders
Source: Eur J Health Econ. 2022 Jul 1;24(4):499–512. doi: 10.1007/s10198-022-01489-z (PMC10175471; doi:10.1007/s10198-022-01489-z)
Supplement: Supplementary file 1 — Supplementary file1 (DOCX 467 KB) [file 10198_2022_1489_MOESM1_ESM.docx]

**Psychometric evaluation of the German version of the Recovering Quality of Life (ReQoL) measures in patients with affective disorders – Supplementary Material**

Additional information on the constructs and scores of the measures used for the assessment of symptom severity and HrQoL

Measures of syptom severity

The Patient Health Questionnaire (PHQ-9) consists of nine items with four levels ranging from ‘not at all’ to ‘nearly every day’. The item scores of the PHQ-9 were summed up to a total score ranging from 0 and 27, with higher scores indicating a higher depressive symptom severity [1, 2]. The Altman Self-Rating Mania Scale (ASRM) consists of five items with five levels. A total score between 0 and 20 can be obtained by summing the item scores, with higher scores indicating a higher severity of manic or hypomanic symptoms [3].

The Global Assessment of Functioning (GAF) is an externally rated scale ranging from 0 (inadequate information) to 100, with 91 to 100 being the highest ratings (superior functioning in a wide range of activities) and 1 to 10 being the lowest ratings (persistent danger of severely hurting self or others, or persistent inability to maintain minimal personal hygiene, or serious suicidal act with clear expectation of death) [4]. The Clinical Global Impression – Severity scale (CGI-S) is also externally rated and its scale consists of seven levels ranging from ‘normal, not at all ill’ ‘among the most extremely ill patients’ [5].

Measures of health-related quality of life

The EQ-5D-5L consists of five items with five levels each. The items address the dimensions of HrQoL ‘mobility’, ‘self-care’, ‘usual activities’, ‘pain/discomfort’, and ‘anxiety/depression’ and are scored on a scale ranging from ‘no problems’ to ‘extreme problems’ [6]. On the basis of 3125 possible health states, an EQ-5D-5L index score was calculated using preference-based value set derived from the German general population [7]. The EQ-5D-5L index score based on the German value set can range from −0.661 (extreme problems in all 5 dimensions) to 1 (no problems in any dimension). An EQ-5D-5L index score of 0 represents a HrQoL that is as bad as death [7]. The EQ-VAS is a visual analogue scale that is part of the EQ-5D-5L for the assessment of subjective HrQoL [8]. The EQ-VAS is able to take values ranging from 0 (worst imaginable health state) to 100 (best imaginable health state) [6].

The SF-12 consists of 12 items with three to five levels that address the 8 dimensions: ‘physical functioning’, ‘physical role limitations’, ‘bodily pain’, ‘general health’, ‘vitality’, ‘social functioning’, ‘emotional role limitations’ and ‘mental health’ [9, 10]. On the basis of the items of the SF-12, an SF-6D index score was calculated using preference-based value set derived from the United Kingdom general population [11]. The SF-6D comprises 6 dimensions of HrQoL by combining the dimension ‘physical role limitations’ with ‘emotional role limitations’ and by excluding the dimension ‘general health’. The SF-6D index score is able to take values from 0 (as bad as death) to 1 (full health).

Furthermore, the mental component summary score (MCS) and physical component summary score (PCS) were calculated from the respective mental and physical dimensions of the SF-12. For this purpose, eight subscales of the SF-12 were calculated by Z-transformation on the basis of norm-based scoring using mean values and standard deviations of a normative sample of the United States population [10]. The MCS and PCS are able to take values from 0 to 100, with higher scores representing better health.


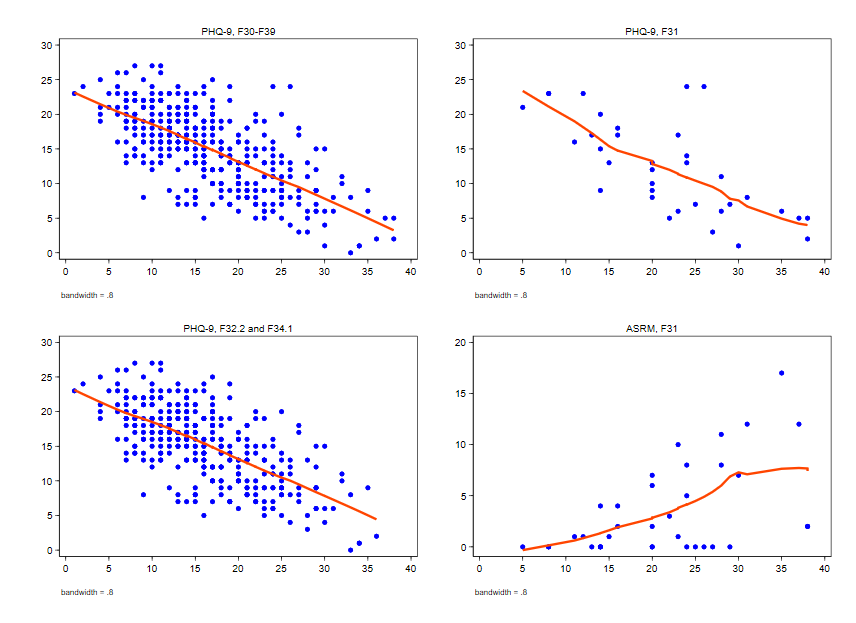


**Figure S1**. Scatterplots and LOWESS curves of the ReQoL-10 and clinical measures (PHQ-9, ASRM) for persons with mood disorders (F30-F39)


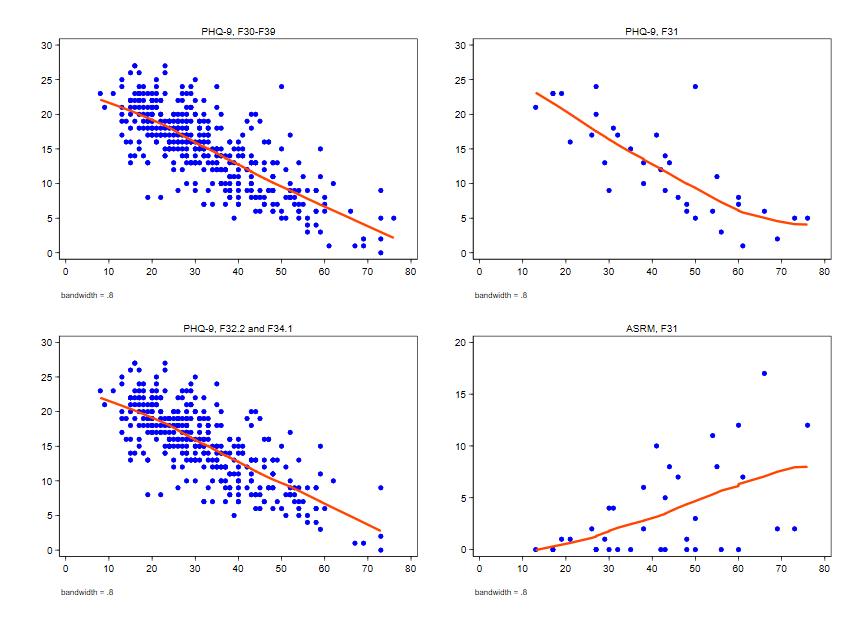


**Figure S2**. Scatterplots and LOWESS curves of the ReQoL-20 and clinical measures (PHQ-9, ASRM) for persons with mood disorders (F30-F39)


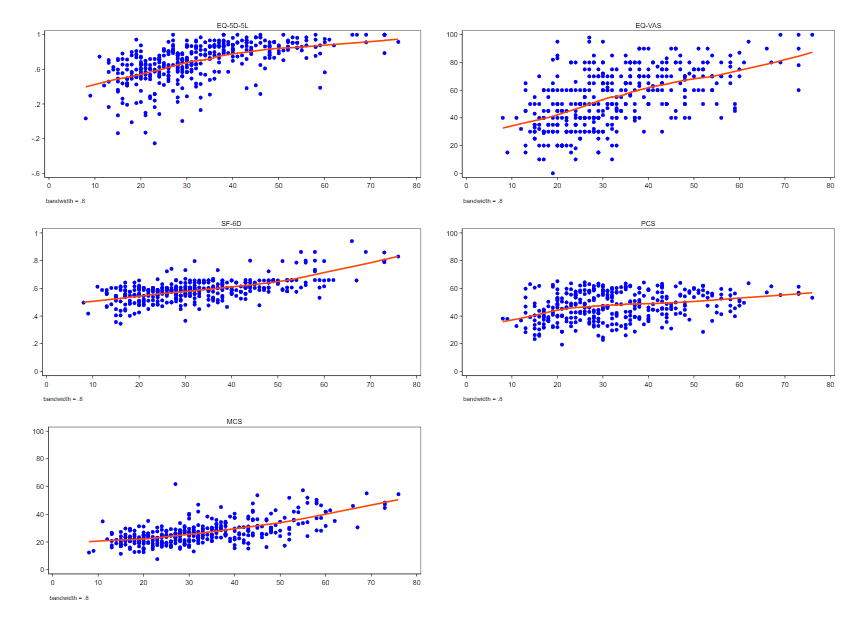


**Figure S3**. Scatterplots and LOWESS curves of the ReQoL-20 and HrQoL-measures (EQ-5D-5L, EQ-VAS, SF-6D, PCS, MCS) for persons with mood disorders (F30-F39)


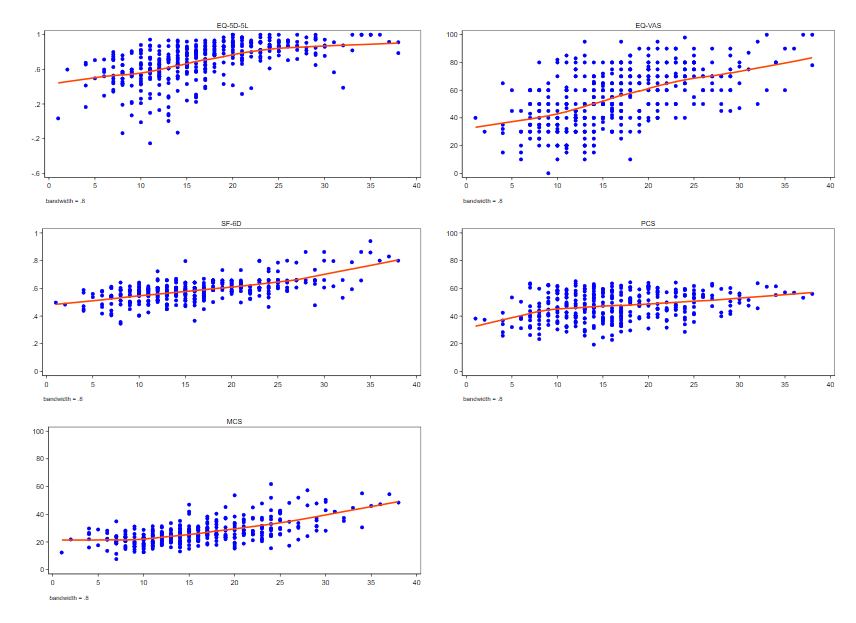


**Figure S4**. Scatterplots and LOWESS curves of the ReQoL-10 and HrQoL-measures (EQ-5D-5L, EQ-VAS, SF-6D, PCS, MCS) for persons with mood disorders (F30-F39)


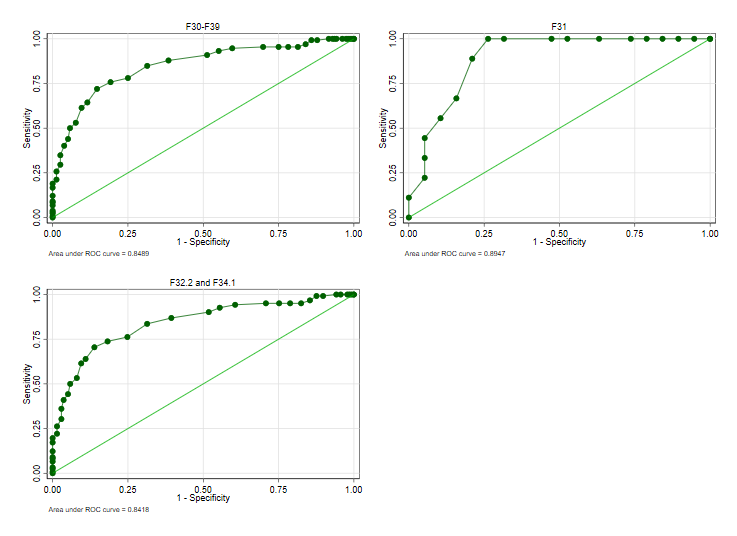


**Figure S3**. ROC curves of the ReQoL-10 difference between T0 and T1 and response based on the clinical measure PHQ-9 for persons with mood disorders (F30-F39; n = 282)


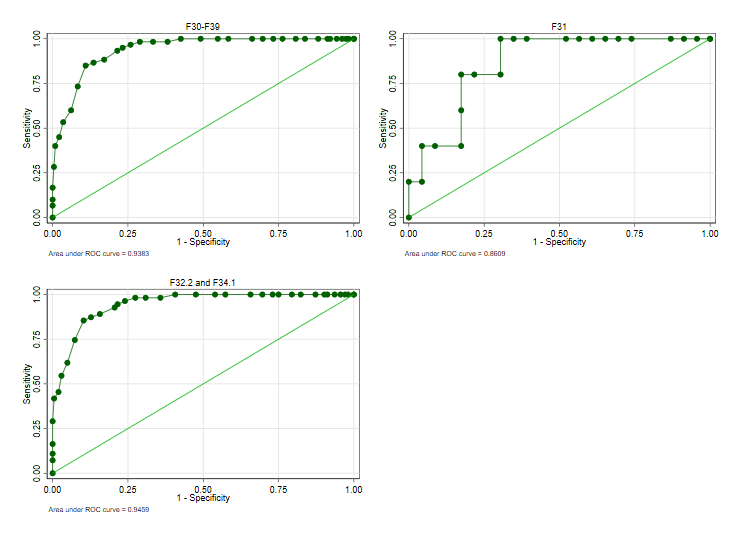


**Figure S4**. ROC curves of the ReQoL-10 at T1 and remission based on the clinical measure PHQ-9 for persons with mood disorders (F30-F39; n = 282)

**Table S1**. Concurrent validity between clinical measures (PHQ-9, ASRM) and measures of health-related quality of life (EQ-5D-5L, EQ-VAS, SF-6D, MCS, PCS) for persons with mood disorders (F30-F39)

| **PCC** | | **All persons with mood disorders (F30-F39; n=393^+^)** | **Persons with bipolar affective disorder (F31; n=36)** | **Persons with major depression and dysthymia (F32.2 and F34.1; n=356)** |
| --- | --- | --- | --- | --- |
| PHQ-9 | EQ-5D-5L (n=374) | 0.58 *** | 0.50 * | 0.58 *** |
|  | EQ-VAS (n=381) | −0.48 *** | −0.62 *** | −0.45 *** |
|  | SF-6D (n=350) | −0.55 *** | −0.49 ** | −0.55 *** |
|  | MCS (n=341) | −0.56 *** | −0.52 ** | −0.56 *** |
|  | PCS (n=341) | −0.17 ** | −0.36 | −0.16 ** |
| ASRM | EQ-5D-5L (n=36) | - | −0.44 * | - |
|  | EQ-VAS (n=36) | - | 0.46 ** | - |
|  | SF-6D (n=29) | - | 0.49 ** | - |
|  | MCS (n=29) | - | 0.39 * | - |
|  | PCS (n=29) | - | 0.21 | - |

^+^ One person had an unspecified mood disorder (F39); PCC: Pearson’s Correlation Coefficient. * *p* ≤ 0.05, ** *p* ≤ 0.01,
*** *p* ≤ 0.001.

**Table S2**. Known-group validity of ReQoL-10/ReQoL-20 using cut-off points of clinical measures (PHQ-9^+^, ASRM^++^) as well as generic measures (CGI-S^+++^, GAF^†^) for persons with mood disorders (F30-F39)

| **ANOVA: Mean difference (SE)** | | **All persons with mood disorders (F30-F39; n=393**^‡^**)** | **Persons with bipolar affective disorder (F31; n=36)** | **Persons with major depression and dysthymia (F32.2 and F34.1; n=356)** |
| --- | --- | --- | --- | --- |
| ReQoL-10 | PHQ-9 (n=383) | 8.03 (0.59)*** | 11.20 (2.38)*** | 7.40 (0.60)*** |
|  | ASRM (n=36) | - | −8.52 (2.91)** | - |
|  | CGI-S (n=393) | 2.64 (0.72)*** | 1.20 (3.50) | 3.98 (0.72)*** |
|  | GAF (n=393) | 2.52 (0.70)*** | 2.67 (3.89) | 3.60 (0.70)*** |
| ReQoL-20 | PHQ-9 (n=383) | 16.97 (1.07)*** | 23.93 (4.24)*** | 15.75 (1.09)*** |
|  | ASRM (n=36) | - | −16.64 (5.60)** | - |
|  | CGI-S (n=393) | 4.71 (1.38)*** | 1.50 (6.76) | 7.21 (1.38)*** |
|  | GAF (n=393) | 4.75 (1.34) *** | 3.30 (7.53) | 6.85 (1.33)*** |

*^+^* PHQ-9 was dichotomized into minimal to moderate depression (PHQ ≤ 14) vs. moderately severe depression to severe depression (PHQ-9 > 14); *^+++^* ASRM was dichotomized into low probability of a manic or hypomanic condition (ASRM < 6) vs. high probability of a manic or hypomanic condition (ASRM ≥ 6); *** The seven original categories of the CGI-S were dichotomized into less severe (1 ≤ CGI-S ≤ 4) vs. more severe illness (4 > CGI-S ≤ 7); † The GAF was dichotomized into good (GAF > 50) vs. poor global functioning (GAF ≤ 50); ‡ One person had an unspecified mood disorder (F39). ANOVA: Analysis of variance, SE: standard error. * *p* ≤ 0.05, ** *p* ≤ 0.01, *** *p* ≤ 0.001.

**Table S3**. Sensitivity to change of ReQoL-10/ReQoL-20 based on the ASRM for persons with bipolar affective disorder (F31) – Response* and Remission**

|  | | **Response** | | | **Remission** | | |
| --- | --- | --- | --- | --- | --- | --- | --- |
|  |  | **N (%)** | **Mean diff. (SD)** | **ES / SRM** | **N (%)** | **Mean diff. (SD)** | **ES / SRM** |
| T0 to T1 | ReQoL-10 (n=29) | 7 (24.14) | −6.43 (10.78) | −1.29 / −0.60 | 21 (72.41) | 2.33 (10.80) | 0.47 / 0.22 |
|  | ReQoL-20 (n=29) |  | −10.86 (20.71) | −2.18 / −0.52 |  | 5.05 (20.87) | 1.01 / 0.24 |
| T1 to T2 | ReQoL-10 (n=28) | 3 (10.71) | −3.00 (1.00) | −0.61 / −3.00 | 22 (78.57) | 0.41 (5.91) | 0.08 / 0.07 |
|  | ReQoL-20 (n=28) |  | −11.33 (6.51) | −2.30 / −1.74 |  | −0.05 (11.09) | −0.01 / −0.00 |
| T0 to T2 | ReQoL-10 (n=29) | 8 (27.59) | −1.13 (8.51) | −0.23 / −0.13 | 22 (75.86) | 4.05 (9.33) | 0.84 / 0.43 |
|  | ReQoL-20 (n=29) |  | −2.50 (15.65) | −0.52 / −0.16 |  | 8.45 (18.81) | 1.75 / 0.45 |

* Response to treatment was defined as reduction of the ASRM by ≥ 4 points; ** Remission of symptoms was defined as an ASRM score of < 5. The effect size was calculated by dividing the mean change on the ReQoL-10/ReQoL-20 by the standard deviation of the ReQoL-10/ReQoL-20 at baseline (Glass' Δ). The standardized response mean was calculated by dividing the mean change on the ReQoL-10/ReQoL-20 by the standard deviation of the change. ES: Effect size; SRM: Standardized response mean.

**Table S4**. Sensitivity to change of measures of HrQoL based on the clinical measure PHQ-9 for persons with mood disorders (F30-F39) – Response*

| **HrQoL- measure** | | **All persons with mood disorders (F30-F39)** | | | **Persons with bipolar affective disorder (F31)** | | | **Persons with major depression and dysthymia (F32.2 and F34.1)** | | |
| --- | --- | --- | --- | --- | --- | --- | --- | --- | --- | --- |
|  |  | **N (%)** | **Mean diff. (SD)** | **ES / SRM** | **N (%)** | **Mean diff. (SD)** | **ES / SRM** | **N (%)** | **Mean diff. (SD)** | **ES / SRM** |
| T0 to T1 | EQ-5D-5L (n=281) | 129 (45.91) | 0.16 (0.23) | 0.69 / 0.69 | 9 (33.33) | 0.10 (0.18) | 0.56 / 0.58 | 119 (47.04) | 0.16 (0.23) | 0.70 / 0.69 |
|  | EQ-VAS (n=290) | 133 (45.86) | 18.31 (19.86) | 0.89 / 0.92 | 9 (33.33) | 18.44 (23.07) | 0.74 / 0.80 | 123 (46.95) | 18.08 (19.64) | 0.89 / 0.92 |
|  | SF-6D (n=263) | 125 (47.53) | 0.11 (0.11) | 1.59 / 1.07 | 8 (38.10) | 0.11 (0.09) | 1.59 / 1.19 | 116 (48.13) | 0.11 (0.11) | 1.57 / 1.06 |
|  | MCS (n=256) | 121 (47.27) | 15.49 (11.37) | 2.24 / 1.36 | 8 (38.10) | 10.74 (12.24) | 0.76 / 0.88 | 112 (47.86) | 15.69 (11.23) | 2.59 / 1.40 |
|  | PCS (n=256) | 121 (47.27) | 0.94 (10.03) | 0.09 / 0.09 | 8 (38.10) | 8.14 (4.30) | 2.55 / 1.89 | 112 (47.86) | 0.44 (10.18) | 0.04 / 0.04 |
| T1 to T2 | EQ-5D-5L (n=260) | 42 (16.15) | 0.15 (0.20) | 0.65 / 0.73 | 3 (11.11) | −0.01 (0.13) | −0.22 / −0.10 | 39 (16.74) | 0.16 (0.20) | 0.71 / 0.79 |
|  | EQ-VAS (n=265) | 42 (15.85) | 17.90 (17.63) | 0.87 / 1.02 | 3 (10.71) | 15.00 (25.98) | 0.79 / 0.58 | 39 (16.46) | 18.14 (17.27) | 0.88 / 1.05 |
|  | SF-6D (n=249) | 41 (16.47) | 0.09 (0.09) | 1.19 / 1.01 | 3 (11.54) | 0.09 (0.16) | 0.64 / 0.58 | 38 (17.04) | 0.09 (0.08) | 1.38 / 1.07 |
|  | MCS (n=245) | 40 (16.33) | 10.48 (9.56) | 1.12 / 1.10 | 3 (11.54) | 12.67 (19.13) | 0.60 / 0.66 | 37 (16.89) | 10.29 (8.80) | 1.26 / 1.17 |
|  | PCS (n=245) | 40 (16.33) | −0.08 (7.00) | −0.01 / −0.01 | 3 (11.54) | −0.16 (2.50) | −0.08 / −0.06 | 37 (16.89) | −0.08 (7.28) | −0.01 / −0.01 |
| T0 to T2 | EQ-5D-5L (n=277) | 130 (46.93) | 0.16 (0.19) | 0.79 / 0.82 | 9 (33.33) | 0.01 (0.37) | 0.08 / 0.04 | 121 (48.40) | 0.17 (0.17) | 0.84 / 0.99 |
|  | EQ-VAS (n=288) | 134 (46.53) | 20.14 (20.19) | 1.00 / 1.00 | 9 (31.03) | 21.44 (27.34) | 0.82 / 0.78 | 125 (48.26) | 20.05 (19.72) | 1.01 / 1.02 |
|  | SF-6D (n=251) | 120 (47.81) | 0.12 (0.11) | 1.72 / 1.09 | 7 (35.00) | 0.07 (0.12) | 1.06 / 0.58 | 113 (48.92) | 0.13 (0.11) | 1.75 / 1.13 |
|  | MCS (n=245) | 117 (47.67) | 16.60 (11.85) | 2.43 / 1.40 | 7 (35.00) | 12.36 (13.73) | 0.84 / 0.90 | 110 (48.89) | 16.87 (11.74) | 2.83 / 1.44 |
|  | PCS (n=245) | 117 (47.67) | 0.26 (9.55) | 0.00 / 0.03 | 7 (35.00) | 3.57 (13.71) | 0.84 / 0.26 | 110 (48.89) | 0.05 (9.27) | 0.00 / 0.01 |

*Response to treatment was defined as reduction of the PHQ-9 by ≥ 5 points. The effect size was calculated by dividing the mean change on the HrQoL-measure by the standard deviation of the HrQoL-measure at baseline (Glass' Δ). The standardized response mean was calculated by dividing the mean change on the HrQoL-measure by the standard deviation of the change. ES: Effect size, SRM: Standardized response mean.

**Table S5**. AUC values of ROC curves of the ReQoL-10 and ReQoL-20 difference between T0 and T1, and response* based on clinical measures (PHQ-9, ASRM) for persons with mood disorders (F30-F39)

| **AUC value (SE)** | | **All persons with mood disorders (F30-F39; n=288)** | **Persons with bipolar affective disorder (F31; n=29)** | | **Persons with major depression and dysthymia (F32.2 and F34.1; n=259)** |
| --- | --- | --- | --- | --- | --- |
|  |  | **PHQ9** | **PHQ-9** | **ASRM** | **PHQ-9** |
| T0 to T1 | ReQoL-10 | 0.85 (0.02) | 0.89 (0.06) | 0.18 (0.09) | 0.84 (0.03) |
|  | ReQoL-20 | 0.88 (0.02) | 0.98 (0.02) | 0.20 (0.11) | 0.87 (0.02) |
| T1 to T2 | ReQoL-10 | 0.85 (0.03) | 0.93 (0.05) | 0.31 (0.08) | 0.85 (0.03) |
|  | ReQoL-20 | 0.87 (0.03) | 0.95 (0.04) | 0.19 (0.11) | 0.86 (0.03) |
| T0 to T2 | ReQoL-10 | 0.84 (0.02) | 0.94 (0.04) | 0.28 (0.10) | 0.83 (0.02) |
|  | ReQoL-20 | 0.89 (0.02) | 0.96 (0.03) | 0.27 (0.12) | 0.88 (0.02) |

* Response to treatment was defined as reduction of the PHQ-9 by ≥ 5 points. AUC: Area under the curve; SE: Standard error.

**Table S6**. Sensitivity to change of measures of HrQoL based on the clinical measure PHQ-9 for persons with mood disorders (F30-F39) – Remission*

| **HrQoL-measure** | | **All persons with mood disorders (F30-F39)** | | | **Persons with bipolar affective disorder (F31)** | | | **Persons with major depression and dysthymia (F32.2 and F34.1)** | | |
| --- | --- | --- | --- | --- | --- | --- | --- | --- | --- | --- |
|  |  | **N (%)** | **Mean diff. (SD)** | **ES / SRM** | **N (%)** | **Mean diff. (SD)** | **ES / SRM** | **N (%)** | **Mean diff. (SD)** | **ES / SRM** |
| T0 to T1 | EQ-5D-5L (n=281) | 58 (20.64) | 0.13 (0.21) | 0.76 / 0.62 | 5 (18.52) | 0.14 (0.24) | 0.60 / 0.56 | 53 (20.95) | 0.13 (0.21) | 0.77 / 0.62 |
|  | EQ-VAS (n=290) | 61 (21.03) | 20.39 (19.69) | 1.05 / 1.04 | 5 (18.52) | 15.80 (25.90) | 0.68 / 0.61 | 56 (21.37) | 20.81 (19.28) | 1.07 / 1.08 |
|  | SF-6D (n=263) | 56 (21.29) | 0.16 (0.12) | 1.86 / 1.30 | 4 (19.05) | 0.15 (0.11) | 2.81 / 1.33 | 52 (21.51) | 0.16 (0.12) | 1.85 / 1.29 |
|  | MCS (n=256) | 53 (20.70) | 19.37 (12.86) | 1.85 / 1.51 | 4 (19.05) | 17.11 (14.24) | 2.27 / 1.20 | 49 (20.94) | 19.56 (12.89) | 1.84 / 1.52 |
|  | PCS (n=256) | 53 (20.70) | 2.58 (9.19) | 0.28 / 0.28 | 4 (19.05) | 9.94 (4.54) | 2.92 / 2.19 | 49 (20.94) | 1.97 (9.24) | 0.21 / 0.21 |
| T1 to T2 | EQ-5D-5L (n=260) | 64 (24.62) | 0.02 (0.09) | 0.17 / 0.20 | 7 (25.93) | 0.02 (0.05) | 0.36 / 0.30 | 57 (24.46) | 0.02 (0.10) | 0.17 / 0.20 |
|  | EQ-VAS (n=265) | 65 (24.53) | 6.55 (12.49) | 0.55 / 0.52 | 7 (25.00) | 6.00 (12.05) | 1.03 / 0.69 | 58 (24.47) | 11.14 (16.05) | 0.49 / 0.50 |
|  | SF-6D (n=249) | 60 (24.10) | 0.05 (0.11) | 0.39 / 0.46 | 6 (23.08) | 0.07 (0.12) | 0.74 / 0.57 | 54 (24.22) | 0.05 (0.11) | 0.37 / 0.44 |
|  | MCS (n=245) | 58 (23.67) | 4.76 (9.85) | 0.43 / 0.48 | 6 (23.08) | 5.48 (14.50) | 0.42 / 0.38 | 52 (23.74) | 4.68 (9.37) | 0.43 / 0.50 |
|  | PCS (n=245) | 58 (23.67) | 0.19 (5.55) | 0.02 / 0.03 | 6 (23.08) | 1.94 (5.54) | 0.28 / 0.35 | 52 (23.74) | -0.01 (5.57) | 0.00 / 0.00 |
| T0 to T2 | EQ-5D-5L (n=377) | 64 (23.10) | 0.13 (0.17) | 0.67 / 0.73 | 7 (25.93) | 0.12 (0.21) | 0.59 / 0.57 | 57 (22.80) | 0.13 (0.17) | 0.67 / 0.75 |
|  | EQ-VAS (n=288) | 66 (22.92) | 20.39 (19.59) | 1.00 / 1.04 | 7 (24.14) | 16.43 (25.05) | 0.67 / 0.66 | 59 (22.78) | 20.86 (19.05) | 1.04 / 1.10 |
|  | SF-6D (n=251) | 60 (23.90) | 0.17 (0.12) | 1.79 / 1.38 | 5 (25.00) | 0.12 (0.16) | 1.27 / 0.75 | 55 (23.81) | 0.12 (0.16) | 1.83 / 1.45 |
|  | MCS (n=245) | 57 (23.27) | 20.31 (12.78) | 1.91 / 1.59 | 5 (25.00) | 8.84 (18.22) | 0.54 / 0.49 | 52 (23.11) | 21.42 (11.80) | 2.24 / 1.81 |
|  | PCS (n=245) | 57 (23.27) | 1.93 (8.22) | 0.21 / 0.23 | 5 (25.00) | 1.40 (8.26) | 1.44 / 1.26 | 52 (23.11) | 7.44 (5.90) | 0.15 / 0.17 |

* Remission of symptoms was defined as a PHQ-9 score of < 5. The effect size was calculated by dividing the mean change on the HrQoL-measure by the standard deviation of the HrQoL-measure at baseline (Glass' Δ). The standardized response mean was calculated by dividing the mean change on the HrQoL-measure by the standard deviation of the change. ES: Effect size, SRM: Standardized response mean.

**Table S7**. AUC values of ROC curves of the ReQoL-10 and ReQoL-20 at T1 and T2, and remission* based on clinical measures (PHQ-9, ASRM) for persons with mood disorders (F30-F39)

| **AUC value (SE)** | | **All persons with mood disorders (F30-F39; n=288)** | **Persons with bipolar affective disorder (F31; n=28)** | | **Persons with major depression and dysthymia (F32.2 and F34.1; n=356)** |
| --- | --- | --- | --- | --- | --- |
|  |  | **PHQ9** | **PHQ-9** | **ASRM** | **PHQ-9** |
| T1 | ReQoL-10 | 0.94 (0.01) | 0.86 (0.08) | 0.38 (0.10) | 0.95 (0.01) |
|  | ReQoL-20 | 0.96 (0.01) | 0.90 (0.06) | 0.38 (0.10) | 0.97 (0.01) |
| T2 | ReQoL-10 | 0.81 (0.03) | 0.71 (0.12) | 0.46 (0.12) | 0.83 (0.03) |
|  | ReQoL-20 | 0.83 (0.03) | 0.73 (0.13) | 0.49 (0.12) | 0.85 (0.03) |

* Remission of symptoms was defined as an PHQ-9 score of < 5. AUC: Area under the curve; SE: Standard error.

References

1. Löwe, B., Spitzer, R., Zipfel, S. & Herzog, W. (2002). *Gesundheitsfragebogen für Patienten (PHQ-D)*. Pfizer GmbH, Karlsruhe

2. Spitzer, R.L., Kroenke, K., Williams, J.B.W. & the Patient Health Questionnaire Primary Care Study Group (1999). Validation and Utility of a Self-report Version of PRIME-MD. The PHQ Primary Care Study. *JAMA*. 282, 1737-1744. <https://doi.org/10.1001/jama.282.18.1737>

3. Altman, E.G., Hedeker, D., Peterson, J.L. & Davis, J.M. (1997). The Altman Self-Rating Mania Scale. *Biol. Psychiatry*. 42, 948-955. <https://doi.org/10.1016/S0006-3223(96)00548-3>

4. Jones, S.H., Thornicroft, G., Coffey, M. & Dunn, G. (1995). A brief mental health outcome scale-reliability and validity of the Global Assessment of Functioning (GAF). *Br. J. Psychiatry*. 166, 654-659. <https://doi.org/10.1192/bjp.166.5.654>

5. Guy, W. (1976). *ECDEU assessment manual for psychopharmacology*. US Department of Health, Education, and Welfare, Public Health Service, Alcohol, Drug Abuse, and Mental Health Administration, National Institute of Mental Health, Psychopharmacology Research Branch, Division of Extramural Research Programs, Rockville, Md.

6. Herdman, M., Gudex, C., Lloyd, A., Janssen, M., Kind, P., Parkin, D., . . . Badia, X. (2011). Development and preliminary testing of the new five-level version of EQ-5D (EQ-5D-5L). *Qual. Life Res.* 20, 1727-1736. <https://doi.org/10.1007/s11136-011-9903-x>

7. Ludwig, K., Graf von der Schulenburg, J.M. & Greiner, W. (2018). German Value Set for the EQ-5D-5L. *Pharmacoeconomics*. 36, 663-674. 10.1007/s40273-018-0615-8

8. The Euroqol Group (1990). EuroQol - a new facility for the measurement of health-related quality of life. *Health Policy*. 16, 199-208

9. Ware, J., Jr., Kosinski, M. & Keller, S.D. (1996). A 12-Item Short-Form Health Survey: construction of scales and preliminary tests of reliability and validity. *Med. Care*. 34, 220-233. <https://doi.org/10.1097/00005650-199603000-00003>

10. Ware, J., Kosinski, M. & Keller, S. (1995). *SF-12: How to Score the SF-12 Physical and Mental Health Summary Scales*. The Health Institute, New England Medical Center, Boston, MA

11. Brazier, J.E. & Roberts, J. (2004). The estimation of a preference-based measure of health from the SF-12. *Med Care*. 42, 851-859. 10.1097/01.mlr.0000135827.18610.0d
